# Sexual and reproductive health (SRH): a key issue in the emergency response to the coronavirus disease (COVID- 19) outbreak

**DOI:** 10.1186/s12978-020-0900-9

**Published:** 2020-04-23

**Authors:** Kun Tang, Junjian Gaoshan, Babatunde Ahonsi

**Affiliations:** 1 School of Medicine, Tsinghua University, Beijing, China; 2 United Nations Population Fund China Office, Beijing, China

**Keywords:** COVID-19, Outbreak, Pandemic, Sexual and reproductive health

## Abstract

The novel coronavirus disease (COVID-19) outbreak was first declared in China in December 2019, and WHO declared the pandemic on 11 March 2020. A fast-rising number of confirmed cases has been observed in all continents, with Europe at the epicentre of the outbreak at this moment.

Sexual and reproductive health (SRH) and rights is a significant public health issue during the epidemics. The novel coronavirus (SARS-CoV-2) is new to humans, and only limited scientific evidence is available to identify the impact of the disease COVID-19 on SRH, including clinical presentation and outcomes of the infection during pregnancy, or for persons with STI/HIV-related immunosuppression. Beyond the clinical scope of SRH, we should not neglect the impacts at the health system level and disruptions or interruptions in regular provision of SRH services, such as pre- and postnatal checks, safe abortion, contraception, HIV/AIDS and sexually transmitted infections. Furthermore, other aspects merit attention such as the potential increase of gender-based violence and domestic abuse, and effects of stigma and discrimination associated with COVID-19 and their effects on SRH clients and health care providers. Therefore, there is an urgent need for the scientific community to generate sound clinical, epidemiological, and psycho-social behavioral links between COVID-19 and SRH and rights outcomes.

## Commentary

On 31 December 2019, the World Health Organization (WHO) was informed of a cluster of cases of pneumonia of unknown cause, first of which reported on 9 December, detected in Wuhan City, Hubei Province of China. The severe acute respiratory syndrome coronavirus 2 (SARS-CoV-2) was identified as the causative virus by Chinese authorities on 7 January, with evidence of human-to-human transmission by 20th January. On 30 January 2020 the outbreak is declared a Public Health Emergency of International Concern (PHEIC) and a pandemic on March 11, 2020.

On March 16, 2020, the total number of cases outside China had overtaken the total number of cases in China. As of 16th March 2020, 167,511 corona virus disease (COVID-19) cases had been confirmed globally in 148 countries. Europe has now become the epicenter of the pandemic. A fast-rising number of confirmed cases has also been observed in many countries, including the US, Iran, Italy and currently counting cases on all continents [1].

As early as in February, the United Nations Population Fund (UNFPA) Asia and Pacific Regional Office issued its first guidance document and elucidated that SRH is a significant public health issue during epidemics [2]. WHO and Human Reproduction Programme (HRP) have also highlighted SRH in its research roadmap for the COVID-19 [3, 4], as part of the overall WHO response to the outbreak.

COVID-19 is new to humans, and only limited scientific evidence is available to identify its impact on sexual and reproductive health (SRH). It is inconclusive as to whether transmission from the pregnant woman to the fetus exists in COVID-19 [5, 6]. Undoubtedly, the risk of mother-to-child transmission, whether during pregnancy, childbirth or breastfeeding, is one of many clinical questions to be answered in relation to modes of transmission. There are other emerging questions that require special attention, for example, whether SARS-CoV-2 disproportionally cause severe illness and death in pregnant and postpartum women, similar to what we observed during other outbreaks causing severe acute respiratory infection (SARI), where evidence raised questions but provided very few answers [7, 8]. Is infection during pregnancy associated with increased risk of fetal demise and medium-to-long-term adverse infant outcomes? Does infection at different stages of pregnancy influence the severity of disease progression and the reproductive outcomes, for example in future pregnancies? The presence and persistence of the virus in bodily fluids including amniotic fluid and breastmilk is still unanswered as well as the underlying risk of sexual transmission or transmission from the woman to the health care provider during delivery.

It is also important to recognise that reproductive health issues may not be restricted to women, but that men may also suffer consequences. SARS-CoV-2 infection may increase the risk of damage to testicular tissues [9], and we are yet unaware of COVID-19 consequences to male SRH. This long-term implication remains to be investigated.

For both men and women, questions around whether persons with STI/HIV-related immunosuppression are at greater risk of contracting COVID-19 and experiencing poorer recovery rates may also be worthy of investigation given their public health implications. An early February 2020 online survey of persons living with HIV in China indicated this issue as a major source of anxiety within this community [10].

Beyond the clinical scope of SRH, we should not neglect the second tier of impact from COVID-19 on SRH. At the health system level, to support Hubei Province’s emergency response, 189 medical teams, consisting of 21,569 health professionals were assembled across China and dispatched to Hubei Province [11]. Although policy directives were issued by the national and provincial health authorities in early February 2020 to protect pregnant women’s access to maternity services [12, 13], the absence of health care workers from their original duty may still have caused interruptions in regular provision of services, such as essential SRH services, including those for pre- and postnatal checks, safe abortion, contraception, HIV/AIDS and sexually transmitted infections.

It has also been suggested that demands of safe abortion services, including information provision, has increased in the hospitals in nearby Hunan Province of China, which may be related to lack of contraceptive commodities or to fear of unknown consequences of infection during pregnancy in the context of an epidemic [14]. These scenarios are similar to what we observed for the Zika and Ebola outbreaks [15], and could be expected during the COVID-19 pandemic across different countries. As was noted during Zika epidemic in Puerto Rico, when quality contraceptive services were made available and accessible, the use of contraceptives to prevent unintended pregnancy and adverse birth outcomes due to prenatal exposure, increased [16]. It would be valuable to track how long it will take for the utilization of essential SRH services to return to their pre-outbreak levels once COVID-19 has become largely controlled. There is evidence that during the 2014–2015 Ebola outbreak in West Africa the utilization of family planning, antenatal care and institutional deliveries declined and did not fully recover to pre-outbreak levels for 6 months [17, 18].

Gender-based and domestic violence is also a major concern of SRH and rights, and the consequences of enforced self-quarantine or compulsory quarantine policy to contain the outbreak are unknown. Evidence shows that quarantine leads to negative psychological effect, including post traumatic stress symptoms, confusion and anger [19]. Quarantine might, therefore, increase the risk of gender-based violence and domestic abuse, which have been observed in other major disease outbreaks [20]. Stigma and discrimination associated with COVID-19, their effects on clients and health care providers, and how these affect the uptake of SRH services should also be of significant research interest judging by lessons learnt from previous epidemics [21].

SRH and rights is a significant public health issue during outbreaks and it should be a priority. As this pandemic is still evolving, it is urgent for the scientific community to generate solid clinical, epidemiological, and psycho-social behavioral links between COVID-19 and SRH and rights outcomes. In particular, there is a strong need for timely planning and actions for epidemiological research and surveillance of the key vulnerable groups of women and adolescents, and to assess the immediate, medium and long-term effects on their SRH. Perhaps more importantly, we need to solidify operational strategies and actions to protect SRH and rights of women, young people, and vulnerable populations during the epidemic. This requires not only the scientists and physicians, but also policy-makers, community organizations, and international agencies, to work in coordination, trust, and solidarity.

## Data Availability

Not applicable.

## Abbreviations

COVID-19: Coronavirus disease
CRF: Case report forms
HIV/AIDS: Human immunodeficiency virus/acquired immunodeficiency syndrome
HRP: UNDP-UNFPA-UNICEF-WHO-World Bank Special Programme of Research, Development and Research Training in Human Reproduction
SARI: Severe acute respiratory infection
SARS-CoV-2: Severe acute respiratory syndrome coronavirus 2
SRH: Sexual and reproductive health
STI/HIV: Sexually-transmitted infection/ human immunodeficiency virus
WHO: World Health Organization
UNFPA: United Nations Population Fund
