# Correction to: Sexual and reproductive health (SRH): a key issue in the emergency response to the coronavirus disease (COVID- 19) outbreak

**DOI:** 10.1186/s12978-020-00926-7

**Published:** 2020-05-26

**Authors:** Kun Tang, Junjian Gaoshan, Babatunde Ahonsi, Moazzam Ali, Mercedes Bonet, Nathalie Broutet, Edna Kara, Caron Kim, Anna Thorson, Soe Soe Thwin

**Affiliations:** 1 School of Medicine, Tsinghua University, Beijing, China; 2 United Nations Population Fund China Office, Beijing, China; 3 UNDP/UNFPA/UNICEF/WHO/World Bank Special Programme of Research, Development and Research Training in Human Reproduction (HRP), Geneva, Switzerland

**Correction to: Reprod Health (2020) 17:59**

**https://doi.org/10.1186/s12978-020-0900-9**

Following publication of the original article [1], we have been notified that there is a mistake in the authors’ team as some authors were not added.

The following authors have to be added:

Moazzam Ali

Mercedes Bonet

Nathalie Broutet

Edna Kara

Caron Kim

Anna Thorson

Soe Soe Thwin

Affiliation of the above-mentioned authors is:

UNDP/UNFPA/UNICEF/WHO/World Bank Special Programme of Research, Development and Research Training in Human Reproduction (HRP), Geneva, Switzerland
